# Prediction of degradation pathways of phenolic compounds in the human gut microbiota through enzyme promiscuity methods

**DOI:** 10.1038/s41540-022-00234-9

**Published:** 2022-07-12

**Authors:** Francesco Balzerani, Daniel Hinojosa-Nogueira, Xabier Cendoya, Telmo Blasco, Sergio Pérez-Burillo, Iñigo Apaolaza, M. Pilar Francino, José Ángel Rufián-Henares, Francisco J. Planes

**Affiliations:** 1 University of Navarra, Tecnun School of Engineering, Manuel de Lardizábal 13, 20018 San Sebastián, Spain; 2 Departamento de Nutrición y Bromatología, Instituto de Nutrición y Tecnología de los Alimentos, Centro de Investigación Biomédica, Universidad de Granada, Granada, Spain; 3 University of Navarra, Biomedical Engineering Center, Campus Universitario, 31009 Pamplona, Navarra Spain; 4 University of Navarra, Instituto de Ciencia de los Datos e Inteligencia Artificial (DATAI), Campus Universitario, 31080 Pamplona, Spain; 5 Area de Genómica y Salud, Fundación para el Fomento de la Investigación Sanitaria y Biomédica de la Comunitat Valenciana-Salud Pública, Valencia, Spain; 6 CIBER en Epidemiología y Salud Pública, Madrid, Spain; 7 Instituto de Investigación Biosanitaria ibs.GRANADA, Universidad de Granada, Granada, Spain

**Keywords:** Biochemical networks, Computational biology and bioinformatics, Physiology

## Abstract

The relevance of phenolic compounds in the human diet has increased in recent years, particularly due to their role as natural antioxidants and chemopreventive agents in different diseases. In the human body, phenolic compounds are mainly metabolized by the gut microbiota; however, their metabolism is not well represented in public databases and existing reconstructions. In a previous work, using different sources of knowledge, bioinformatic and modelling tools, we developed AGREDA, an extended metabolic network more amenable to analyze the interaction of the human gut microbiota with diet. Despite the substantial improvement achieved by AGREDA, it was not sufficient to represent the diverse metabolic space of phenolic compounds. In this article, we make use of an enzyme promiscuity approach to complete further the metabolism of phenolic compounds in the human gut microbiota. In particular, we apply RetroPath RL, a previously developed approach based on Monte Carlo Tree Search strategy reinforcement learning, in order to predict the degradation pathways of compounds present in Phenol-Explorer, the largest database of phenolic compounds in the literature. Reactions predicted by RetroPath RL were integrated with AGREDA, leading to a more complete version of the human gut microbiota metabolic network. We assess the impact of our improvements in the metabolic processing of various foods, finding previously undetected connections with output microbial metabolites. By means of untargeted metabolomics data, we present in vitro experimental validation for output microbial metabolites released in the fermentation of lentils with feces of children representing different clinical conditions.

## Introduction

Phenolic compounds are products of the secondary metabolism of plants^1^, produced by synthesis through the pentose phosphate, shikimate and phenylpropanoid pathways^2^. Their structure consists of benzene rings with one or more hydroxyl groups, and they can be simple phenolic molecules (i.e. phenolic acids) or be highly polymerized in complex compounds (i.e. flavonoids or tannins)^3,4^. Phenolic compounds are the most abundant natural antioxidants present in the human diet, and are found in large amounts in foods of plant origin, including fruits and plant-derived beverages^2,4–6^.

There is an increasing body of evidence supporting that phenolic compounds are potent antioxidants and limit the risk of several diseases to which oxidative damage is a significant contributor^4,6,7^. In particular, it is well established that introducing some polyphenols with the diet or as supplements can improve the health status of people affected by cardiovascular disease, and this is confirmed by several biomarkers associated to this condition and by epidemiologic studies^5,7^. For instance, it has been indicated that a high flavonoid intake is related to a lower mortality from coronary heart disease and a lower incidence of myocardial infarction in older men^8^. In addition, a high dietary flavonoid intake can reduce the risk of coronary heart disease by 38% in postmenopausal women^8^. Similar studies about the role of phenolic compounds in other major diseases, such as cancer, diabetes and obesity, are growing and increasing the evidence for the beneficial effects of polyphenols derived from plants for human health^4,6,8–11^.

Due to their complex chemical structures, high molecular-weight polyphenols are not easily absorbed in the small intestine and reach the colon almost unchanged^12^. In the intestinal lumen area, the microbiota helps to break down these complex molecules into absorbable phenolic metabolites and increases the biological availability of polyphenols through their conversion into smaller and more active compounds^12^. Therefore, the gut microbiota exerts a major function in the bioavailability and bioactivity of polyphenols, which has a direct influence on human health, and modifications to the composition of the former affect the availability of the latter^12^. This interaction is quickly becoming a major research topic in the area of personalized nutrition^13,14^.

The metabolism of phenolic compounds in the human gut microbiota remains largely unknown. Universal metabolic databases, such as KEGG^15^ or the Model SEED database^16^, store reactions from species not present in the gut microbiota, and pathway extraction is not direct. In a previous work^17^, we addressed this issue and developed AGREDA^17^, an extension of AGORA^18^, the most comprehensive collection of metabolic reconstructions for the human gut microbiota. AGREDA^17^ provides a better description of the metabolic pathways of dietary compounds, including 114 phenolic compounds of Phenol-Explorer^19^, the largest database of phenolic compounds in the literature. However, there is still substantial room for improvement. In particular, more than 2/3 of the phenolic compounds present in Phenol-Explorer^19^ are not even described in universal metabolic databases, which makes the definition of their metabolic pathways more challenging, requiring the use of different approaches.

Here, we rely on enzyme promiscuity to complete metabolic pathways of phenolic compounds in the human gut microbiota. Enzyme promiscuity assumes that enzymes could accept alternative substrates and catalyze additional reactions to the ones annotated in databases^20–23^, typically referred to as the underground metabolism^24,25^. Several algorithms have been developed to exploit the concept of enzyme promiscuity and predict synthesis/degradation pathways for metabolites absent in universal databases^21–23,26,27^. They extract reaction rules from known enzymatic reactions, and use them to describe potential structural changes in the bonding patterns of substrates and products^27^. Reaction rules are defined to be as generic as possible, so that they can be applied to different substrates to establish potential unknown reactions. Possible transformations from the reaction rules define the so-called extended metabolic network, which typically suffers from combinatorial explosion^27^. Various algorithms address this issue by ranking tentative reactions and metabolites and adopting an appropriate search procedure to infer the most relevant pathways^23,27^. Here, we used RetroPath RL^27^, a recently released open-source Python package, based on Monte Carlo Tree Search strategy reinforcement learning, which significantly improves previous approaches developed by the same group^28,29^.

Using RetroPath RL^27^, we analyzed tentative metabolic pathways for the phenolic compounds present in Phenol-Explorer^19^. We provide details as to the reactions, metabolites and species involved in the proposed pathways and evaluate their chemical and biological plausibility. Then, we integrate these predicted reactions with our previous metabolic reconstruction of the human gut microbiota, AGREDA^17^, and systematically analyze the metabolic capabilities acquired in the extended reconstruction. We assess the impact of our improvements in the metabolic processing of various foods detailed in the Phenol-Explorer database^19^, finding previously undetected connections with output microbial metabolites. By means of untargeted metabolomics data, we present experimental in vitro validation for output microbial metabolites released in the fermentation of lentils with feces of children representing different clinical conditions.

## Results

### Construction of AGREDA_1.1

In a previous work, we developed AGREDA^17^, a metabolic network of the human gut microbiota that more accurately describes the degradation pathways of dietary compounds, including 114 phenolic compounds from Phenol-Explorer^19^. Our objective here is to extend AGREDA^17^ and fill gaps for the remaining 258 phenolic compounds present in Phenol-Explorer^19^ via enzyme promiscuity. Enzyme promiscuity methods extend the metabolic space by considering that enzymes can accept substrates other than those present in annotated reactions. Here, we used RetroPath RL^27^, one of the most advanced retrosynthesis algorithms in the literature that is based on Monte Carlo Tree Search strategy reinforcement learning^30^.

**Fig. 1:**
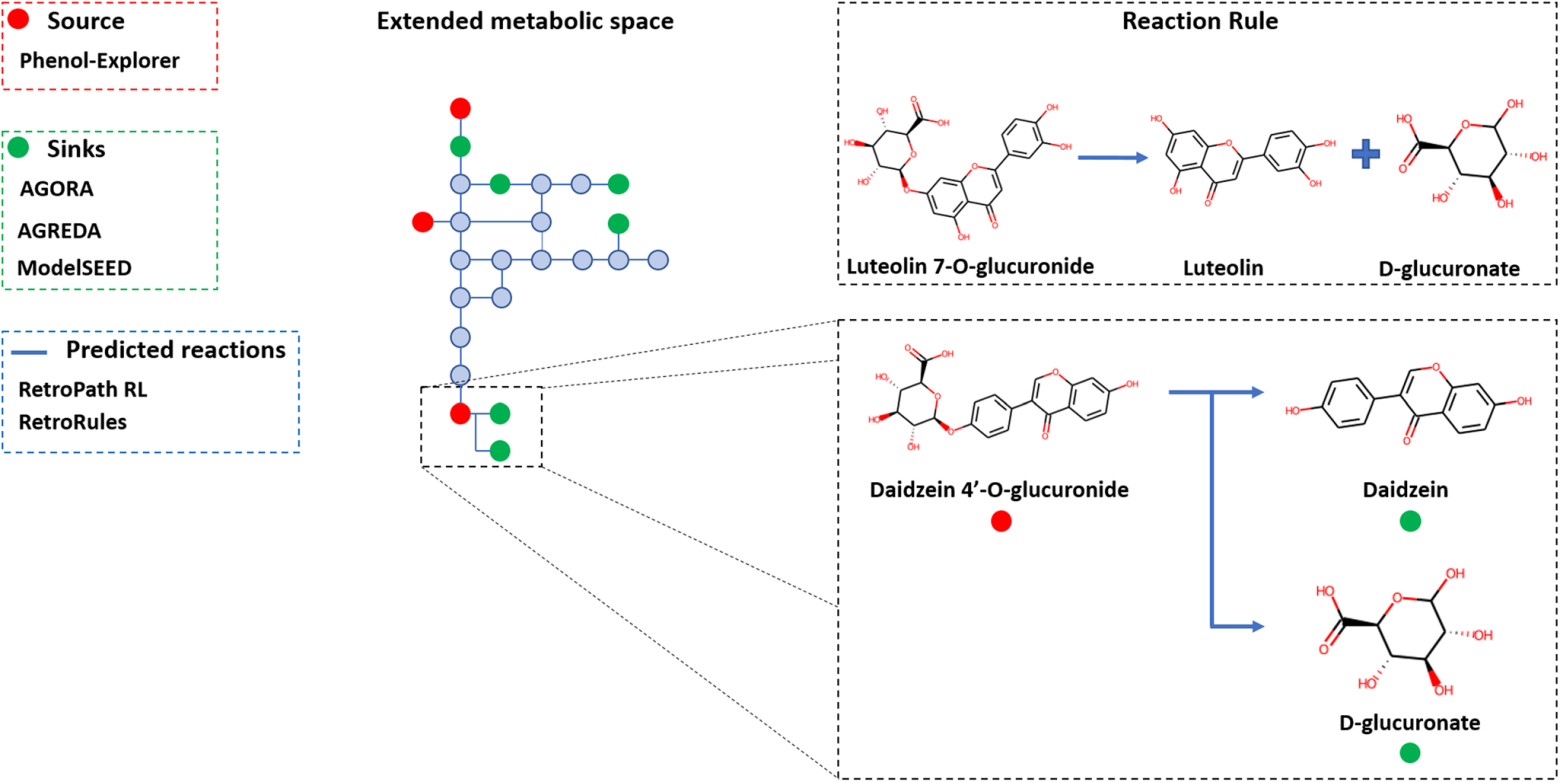
Summary of the enzyme promiscuity pipeline. The extended metabolic space analysis connects sources to sinks through reactions inferred by RetroPath RL^27^ with rules derived from RetroRules^34^. Sources are phenolic compounds obtained from Phenol-Explorer^19^ (colored red), and sinks are metabolites involved in reactions existing in species present in AGORA^18^ (colored green), which were obtained from AGREDA^17^ and the Model Seed Database^16^. An example predicted reaction by the RetroPath algorithm^27^ is shown. This reaction transforms the source *Daidzein 4’-O-glucuronide* into *Daidzein* and *D-glucuronate*, using a rule derived from the annotated reaction that produces *Luteolin* and *D-glucuronate* from *Luteolin 7-O-glucuronide*. 2D chemical structures were drawn using RDKit^36^.

RetroPath RL^27^ requires three different input data: sources, sinks and reaction rules. Sources are phenolic compounds obtained from Phenol-Explorer^19^, and sinks are metabolites involved in reactions existing in species present in AGORA^18^ (colored green). These metabolites were obtained from AGREDA^17^ and the Model Seed Database^16^ (see Methods section). Reaction rules are generic structural representations of reactions and define chemical transformations that can potentially occur. As with metabolites, we only considered rules coming from reactions annotated to the species present in AGORA^18^ and, thus, in the human gut microbiota. RetroPath RL^27^ searches for paths that link source and sink metabolites through the extended metabolic space derived from reaction rules. The steps that were followed to apply RetroPath RL^27^ are detailed below and summarized in Fig. 1.

We applied RetroPath RL^27^ to the 372 compounds present in the Phenol-Explorer database^19^. We found putative degradation pathways for 303 phenolic compounds. In particular, these pathways involved 191 phenolic compounds that were not previously described in AGREDA^17^. 86 phenolic compounds out of these 191 were connected to the subset of sink metabolites. The remainder 105 phenolic compounds were linked to metabolites that are not included in our metabolic database and, thus, were discarded for further analysis. Full details of reactions and metabolites predicted by RetroPath RL^27^ can be found in Supplementary Data 1.

In order to validate the results derived from RetroPath RL^27^, we assessed the predicted reactions for phenolic compounds that were already present in AGREDA^17^ (112 out of 303 compounds). First, we found that 52.8% of these predicted reactions were part of AGREDA^17^. Moreover, for 92.7% of these 112 phenolic compounds, RetroPath RL^27^ predicted at least one reaction that was already in AGREDA^17^, meaning that for each metabolite the algorithm reaches known transformations and proposes new additional reactions. These results permitted us to be confident that the RetroPath RL^27^ workflow is able to reach correct transformations.

Then, we integrated the reactions and metabolites predicted by RetroPath RL^27^ with AGREDA^17^, following the gap-filling process and single-species analysis described in the Methods section, leading to a new version of the human gut microbiota metabolic network: AGREDA_1.1. To facilitate the comparison, our previous version of AGREDA is referred to as AGREDA_1.0^17^. Overall, AGREDA_1.1 included 133 new metabolites, with 80 new input phenolic compounds, and 313 new reactions with respect to AGREDA_1.0^17^, with 195 reactions predicted by RetroPath RL^27^, obtaining a final network comprising 2735 metabolites and 6257 reactions. Note here that, as in AGREDA_1.0^17^, all reactions added in AGREDA_1.1 have taxonomic annotations to species present in AGORA^18^. Full details of AGREDA_1.1 can be found in Supplementary Data 2.

**Fig. 2:**
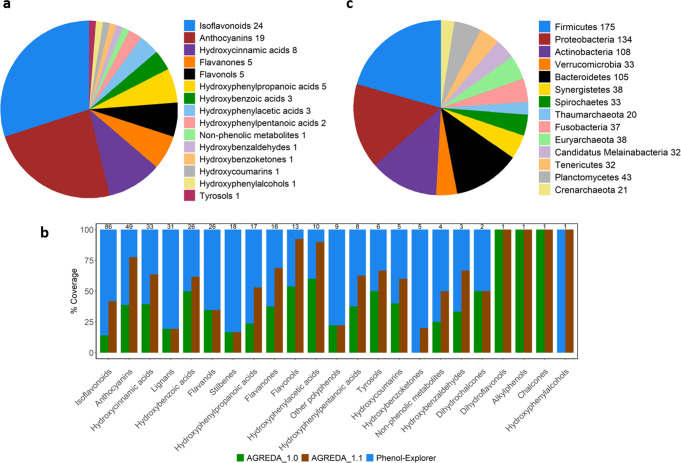
Main metabolic features included in AGREDA_1.1. **a** Representation of the different sub-classes of input phenolic compounds added to AGREDA_1.1. The number of compounds captured by AGREDA_1.1 for each sub-class is detailed in the legend, e.g. ‘Isoflavanoids 24’; (**b**) Barplot showing the percentage coverage of AGREDA_1.0 and AGREDA_1.1 in terms of phenolic compounds included in Phenol-Explorer^19^. The number at the top of the bars is the total number of phenolic compounds for each sub-class, e.g. 86 compounds for Isoflavanoids; (**c**) Contribution of different phyla to the reactions added to AGREDA_1.1. The number of reactions added to AGREDA_1.1 present in each phylum is also provided in the legend, e.g. ‘Firmicutes 175’. Source Data are provided as a Source Data file.

Input phenolic compounds included in AGREDA_1.1 belong to 15 different sub-classes. In particular, we were able to considerably improve the description of three large sub-classes: anthocyanins, isoflavonoids and hydroxycinnamic acids (Fig. 2a). The difference in coverage of PhenolExplorer^19^ compounds between AGREDA_1.0 and AGREDA_1.1 was 28% for isoflavonoids (12 vs 36 out of 86 compounds), 39% for anthocyanins (19 vs 38 out of 49 compounds) and 24% for hydroxycinnamic acids (13 vs 21 out of 33 compounds) (Fig. 2b). On the other hand, all major phyla of the human gut microbiota, i.e. Firmicutes, Bacteroidetes, Proteobacteria and Actinobacteria, were involved in the degradation of these phenolic compounds (Fig. 2c).

### Functional analysis of foods in Phenol-Explorer with AGREDA_1.1

We assessed the relevance of input phenolic compounds added to AGREDA_1.1 using again the Phenol-Explorer database^19^, which details the nutritional composition for 458 foods. We identified 40 foods that involve at least one of the 80 phenolic compounds included in AGREDA_1.1 in their nutritional composition (Supplementary Table 1). Specifically, AGREDA_1.1 improved the representation of the foods by 2.2 phenolic compounds per food on average, with a maximum of 10 and a minimum of 1. This allowed us to describe a wide range of foods more completely, including coffee beverages, fruits, juices, jams, and vegetables.

**Fig. 3:**
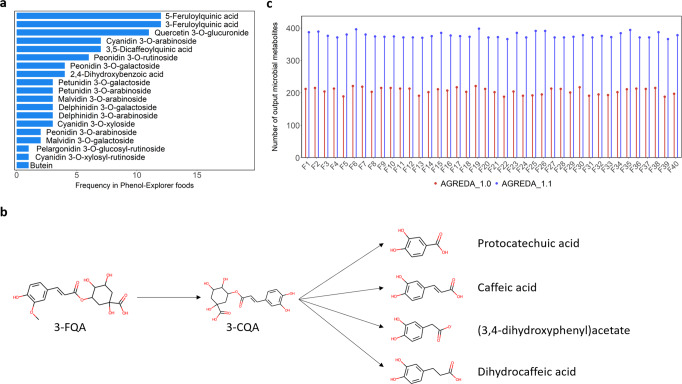
Functional analysis of AGREDA_1.1 with foods available in Phenol-Explorer19. **a** Frequency of input phenolic compounds added to AGREDA_1.1 in the 40 foods of Phenol-Explorer^19^ (F1, F2, …, F40); (**b**) Degradation pathway of 3-Feruloylquinic acid (3-FQA) predicted by AGREDA_1.1. 2D chemical structures were drawn using RDKit^36^; (**c**) Number of output microbial metabolites derived from the input compounds available in the 40 foods of Phenol-Explorer^19^ with AGREDA_1.0^17^ and AGREDA_1.1. Source Data are provided as a Source Data file.

Figure 3a shows the subset of phenolic compounds added to AGREDA_1.1 that takes part in the 40 recipes considered. The most frequent compounds are 3-Feruloylquinic acid (3FQA) and 5Feruloylquinic acid (5FQA). 3FQA and 5FQA constitute a source of ferulate, which can be converted into different bioactive molecules. However, we also predicted their demethylation into 3-Caffeoylquinic acid (3CQA) and 5-Caffeoylquinic acid (5CQA), respectively, as previously hypothesized in other works, due to the low levels of 3FQA and 5FQA observed in plasma^31^ (Fig. 3b). In the foods analyzed, 3CQA and 5CQA could not be reached with the previous version of AGREDA^17^, and, thus, their output microbial metabolites were neglected. This same pattern is observed in the degradation of several input phenolic compounds added to AGREDA_1.1, as discussed in detail below.

Note here that only 18 out of 80 input phenolic compounds added to AGREDA_1.1 participated in the foods analyzed with Phenol-Explorer^19^. This does not mean that the remainder 62 phenolic compounds are irrelevant. According to Phenol-Explorer^19^, they are metabolites identified in urine and/or plasma in different experimental studies; however, they were not considered in the nutrient composition analysis of foods. They are associated with relevant nutritional supplements, such as soy milk or red glover supplements (Supplementary Table 2), and, in many cases, they are conjugated polyphenol metabolites with insufficient evidence in the literature. Moreover, we checked that all of these metabolites can be produced as output microbial metabolites from other added input metabolites in AGREDA_1.1, in line with the observations found in Phenol-Explorer^19^.

For each of the 40 foods considered, assuming that all species in AGREDA^17^ take part of the community model, we analyzed the number of output microbial compounds that can be potentially derived from the input phenolic compounds present in AGREDA_1.0^17^ and AGREDA_1.1 using Flux Variability Analysis (FVA)^32^ (see Methods section). On average, AGREDA_1.1 predicted 172 output compounds that were not captured by AGREDA_1.0^17^, with a minimum of 158 and a maximum of 199. Full details can be found in Fig. 3c, which shows the number of output metabolites predicted by AGREDA_1.0^17^ and AGREDA_1.1 for the foods analyzed. All the output microbial metabolites reached using AGREDA_1.0^17^ were present in the ones obtained with AGREDA_1.1. Moreover, the output metabolites we reached with the new reconstruction included some that were not produced with AGREDA_1.0^17^, but were part of the original network (see Fig. 3b), with an average of 135 exchanges, a maximum of 154 and a minimum of 123. This means that the knowledge introduced with this study connected the new phenolic compounds properly, generating the possibility to activate some fluxes that were previously blocked.

### Functional analysis of in vitro lentil fermentation with AGREDA_1.1

We conducted an analysis similar to the one in our previous work^17^ and compared the different microbial output metabolites predicted by the two versions of AGREDA for in vitro fermentation of lentils using 24 children’s fecal samples representing four different clinical conditions, i.e. lean, obese, allergic to cow’s milk and celiac (see Methods section). We contextualized each version of AGREDA with the nutritional composition of lentils and the information of the microbial community of each fecal inoculum obtained from 16 S rRNA gene sequencing data (further details in Supplementary Tables 3–4), obtaining 24 context-specific AGREDA_1.0^17^ models and 24 context-specific AGREDA_1.1 models, and predicted the potential list of byproducts that can be derived in each specific condition via FVA^32^ (see Methods section). We validated the results by means of an untargeted metabolomics approach (see Methods section).

**Fig. 4:**
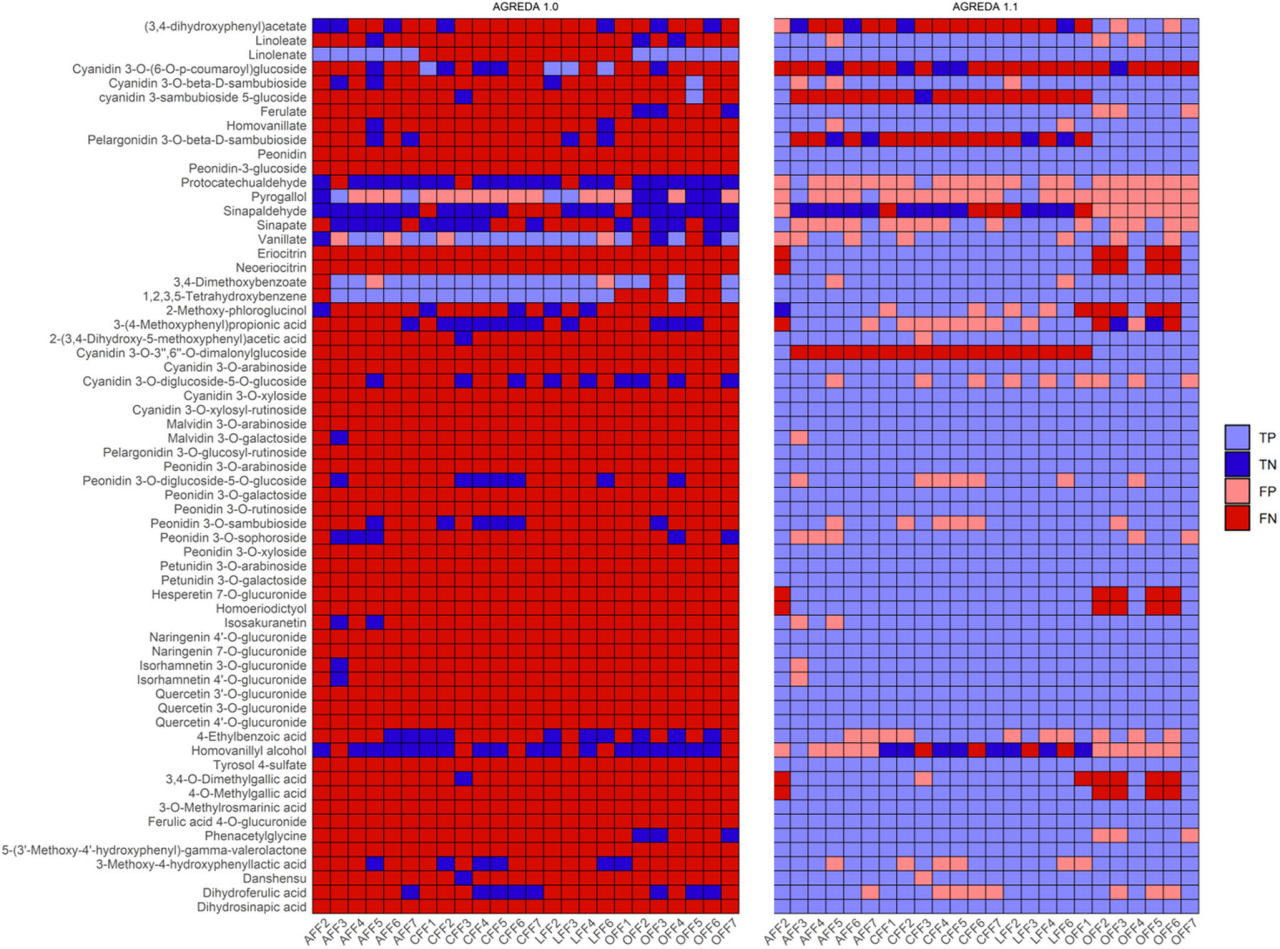
Comparison between the predictions of AGREDA_1.017 and AGREDA_1.1 with in vitro experiments. Representation of the presence of 63 output microbial compounds predicted in AGREDA_1.0^17^ and AGREDA_1.1 to derive from the fermentation of lentils with children feces and measured with an untargeted metabolomics approach. “AFF2”, “AFF3”, “AFF4”, “AFF5”, “AFF6” and “AFF7” denote samples 2, 3, 4, 5, 6 and 7 from children allergic to cow’s milk, respectively; “CFF1”, “CFF2”, “CFF3”, “CFF4”, “CFF5”, “CFF6” and “CFF7” denote samples 1, 2, 3, 4, 5, 6 and 7 from celiac children, respectively; “LFF2”, “LFF3”, “LFF4” and “LFF6” denote samples 2, 3, 4 and 6 from lean children, respectively; “OFF1”, “OFF2”, “OFF3”, “OFF4”, “OFF5”, “OFF6” and “OFF7” denote samples 1, 2, 3, 4, 5, 6 and 7 from obese children, respectively; TP true positives, TN true negatives, FP false positives, FN false negatives. Source Data are provided as a Source Data file.

In particular, we focused on output microbial metabolites with a different predicted result between AGREDA_1.0^17^ and AGREDA_1.1 in at least one of the 24 samples considered. We identified a total number of 63 metabolites that presented differences between the two models. Results obtained from the metabolomics data for the 63 metabolites accross the 24 samples can be found in Fig. 4 (further details in Supplementary Table 5). We found a significant relationship between the predicted metabolites and the in vitro metabolomics data for both metabolic models, but we improved considerably the *p* value of the association in AGREDA_1.1 (two-sided Fisher test *p* value: 0.00094 vs 0.02, respectively; Fig. 4). We can therefore conclude that the newly elucidated compounds and associated metabolic pathways remarkably improve our undestarding of the human gut microbiota metabolism and allow us to predict microbial-derived byproducts that are not considered in the current state of the art.

## Discussion

Phenolic compound metabolism mainly takes place in the gut microbiota and the associated output metabolites have been shown to be beneficial for the health of people affected by different diseases. This fact has attracted the interest of researchers in developing methods that predict output metabolites that can be derived from different input phenolic compounds in the human gut microbiota. Constraint-based modeling, driven by genome-scale metabolic networks, constitutes a promising strategy to address this question.

However, current metabolic reconstructions of the human gut microbiota only partially detail the metabolism of phenolic compounds, which limits the application of constraint-based modeling approaches. In a previous work, we substantially improved the coverage of degradation pathways of phenolic compounds in the human gut microbiota and integrated them with AGORA^18^, obtaining a more complete reconstruction called AGREDA^17^. Using this knowledge base, in this article we use an enzyme promiscuity approach to complete further the metabolism of polyphenols in the human gut microbiota.

Enzyme promiscuity refers to the ability of enzymes to accept different substrates and conduct different chemical transformations to the ones annotated in metabolic databases^33^. In recent years, several models have been developed to assess the application of enzyme promiscuity. The present study applies the RetroPath RL^27^ algorithm that uses a Monte Carlo Tree Search strategy of reinforcement learning to predict putative reactions related to the molecules of interest. RetroPath RL^27^ is one of the most advanced retrosynthesis algorithms in the literature, which improves previous approaches developed by the same group^28,29^.

The RetroPath RL^27^ workflow was applied to predict in the human gut microbiota the metabolic space of the phenolic compounds available in Phenol-Explorer^19^, the largest database of phenolic compounds in the literature. RetroPath RL^27^ found degradation routes for approximately 200 compounds that were not part of previous reconstructions; however, we could only reliably integrate 80 of these phenolic compounds with the AGREDA reconstruction^17^, leading to an updated version of the metabolic network of the human gut microbiota, termed AGREDA_1.1. In this process, we applied the same bioinformatic tools employed in the construction of AGREDA^17^, adding 133 metabolites and 313 reactions to the metabolic network. Moreover, we conducted different quality checks to guarantee a high level of confidence in the predicted reactions: significant recovery of previously annotated reactions with RetroPath RL^27^, taxonomic annotation to species in the human gut microbiota, intermediate metabolites annotated to chemical databases, mass balance and manual curation.

Even though we improved the representation of the phenolic compounds of Phenol-Explorer^19^ notably (as shown in Fig. 2b), we are still far from the complete coverage of the database. Other techniques may need to be considered in order to gain a better understanding of this particular region of the gut microbiota’s metabolic space, whether that comes in the form of a new algorithm that exploits enzyme promiscuity or some other literature sources to extend the metabolic space.

In addition, our predicted reactions enhance the representation of the foods from Phenol-Explorer^19^ in the metabolic network, increasing the number of inputs and outputs that can be associated with the composition of foods. Interestingly, the new subset of input phenolic compounds added to AGREDA_1.1 allows us to reach output microbial compounds that were not possible with AGREDA_1.0^17^ in the different foods analyzed. The biological relevance of these output microbial metabolites was confirmed with the untargeted metabolomics data, obtained from lentils fermentation with feces of children representing different clinical conditions.

Despite these positive results in the lentils fermentation study, we found a high number of false positives for few predicted output metabolites, e.g. protocatechualdehyde (see Fig. 4). This limitation is due to the under-determination in flux prediction in genome-scale metabolic models, but it does not invalidate the predicted metabolic pathways with RetroPath RL^27^. Our predictive computational approach, which considers that an output metabolite is not present in the sample if the maximum flux through its exchange reaction is zero, could be little restrictive for certain metabolic pathways (see Supplementary Fig. 1). The availability of meta-transcriptomics and meta-proteomics data would be very informative to break this under-determination and increase the accuracy of our predictive approach.

In our opinion, enzyme promiscuity and computational prediction algorithms can improve and accelerate the description of the human metabolism and the mutual relationship between human gut microbiota and diet, namely by introducing predicted pathways of important nutritional compounds that have not yet been characterized. The proposed methodology and the AGREDA_1.1 metabolic network presented in this article can drive further the representation of relevant classes of compounds within the diet further, increasing the accuracy of personalized nutrition approaches.

## Methods

### Enzyme promiscuity analysis with RetroPath RL^27^

The RetroPath RL algorithm^27^, a tool developed in Python and executable through the UNIX shell, requires three different input data. In order to generate them, we first built a metabolic database of reactions that are potentially present in the human gut microbiota. In a previous work^17^, we constructed a universal database by merging AGORA^18^ and the Model SEED database^16^. Here, we also included reactions available in the RetroRules database^34^, specifically designed to work with retrosynthesis algorithms. We kept reactions with taxonomic evidence to species present in AGORA^18^ and with available InChI (IUPAC International Chemical Identifier) identifiers for their associated metabolites, as required by RetroPath RL^27^. We obtained 9846 reactions and 6382 metabolites.

We used two approaches to obtain the InChI identifiers for metabolites. On the one hand, we used the KEGG database^15^ and the HMDB database^35^, from which the InChI ID, the molecular structures in MOL files or the SMILES string were extracted. Where necessary, we then used RDKit^36^ to turn these structures or SMILES into InChI strings. On the other hand, we used the Phenol-Explorer database^19^ to get the InChI strings directly for phenolic compounds.

#### Input data for RetroPath RL^27^

RetroPath RL^27^ distinguishes between sink and source metabolites. In our case, source metabolites are those present in the Phenol-Explorer database^19^ (372 compounds) and sink metabolites are those present in the metabolic database described above (6382 compounds). We introduced the InChI identifiers of the compounds in the source and target set into RetroPath RL^27^.

In addition, RetroPath RL^27^ needs reaction rules, which constitute generic representations of reactions and their underlying structural changes in bonding patterns. In particular, RetroPath RL^27^ requires the rules in the community-standard SMARTS (SMILES arbitrary target specification) formalism. We extracted them from the RetroRules database^34^, where they are defined with different levels of specificity depending on the atom distance to the reaction center (reaction diameter). In addition, we manually generated the rules for a set of 236 reactions present in AGREDA^17^, which were previously extracted from the literature and involve specifically other phenolic compounds. The creation of the rules was carried out using the online rule generator present in the RetroRules^34^ website. Once we discarded reaction rules without taxonomic evidence to species present in AGORA^18^, we introduced a total of 49498 reaction rules into RetroPath RL^27^.

#### Parameters of RetroPath RL^27^

Once the sources, sinks and reaction rules were defined, we adjusted various parameters available in RetroPath RL^27^. First, we fixed the biosensor setting, which specifically searches for pathways that connect unknown compounds of interest (sources) to target compounds (sinks)^26,27^. In addition, following the recommendations of RetroPath RL^27^, we considered reaction diameters from 6 to 16 to control the level of promiscuity in the extended metabolic space. Moreover, the internal cut off scores of RetroPath RL^27^, biological and chemical, were fixed to 0.1 and 0.6, respectively, in order to maintain a balance that would neither be too restrictive, nor would it compare molecules that were too dissimilar (see Supplementary Fig. 2 for further details). Finally, RetroPath RL^27^ provides several parameters to terminate the search process. Here, for each phenolic compound, we fixed a maximum number of iterations, itermax = 1000, and computation time limit, time_budget = 28,800 s.

#### Analysis of RetroPath RL^27^ results

We applied RetroPath RL^27^ in the conditions described above to each polyphenol present in the source set. RetroPath RL^27^ returns full scope output, which presents different predicted pathways of the source compound under study. The predicted pathways could be disconnected from our metabolic database. This occurs when their target (end) metabolite is not present in the sink set once the maximum number of iterations and/or the time limit described above is reached. To address this issue, we selected pathways that are connected to our metabolic database. This task was done in an automatic manner for each source compound under study.

At the end of this process, we manually curated the results of the whole workflow. Since RetroPath RL^27^ works with mono-substrates rules, we needed to study the predicted equations in order to have balanced molecular components and atoms. Hence, we extracted the template reactions that RetroPath RL^27^ used to propose the new predicted reactions and we analyzed the chemical structure of the equations, adding the missing substrates (see Supplementary Note 1). Furthermore, we applied the python ChemPy^37^ package to balance the new equations at the atomic level and obtain the stoichiometry of the reactions. With this workflow, we obtained 292 predicted reactions for a total of 86 phenolic compounds and 64 predicted metabolites.

### Update of the AGREDA reconstruction

In order to integrate the phenolic compounds into the AGREDA reconstruction^17^, we first added the predicted reactions and metabolites obtained from the RetroPath RL^27^ workflow into the universal database used in that work. This universal database contains all the reactions in AGORA^18^, the Model SEED database^16^ and literature knowledge, including their taxonomic annotation to the species in AGORA^18^ (present in the human gut microbiota) and functional annotation (EC number).

Then, we applied the same gap filling strategy as the one implemented in the AGREDA reconstruction^17^. This step is necessary because predicted reactions from RetroPath RL^27^ may connect to metabolites present in the universal database but not in AGREDA^17^. The connection to AGREDA^17^ is done by minimizing the inclusion of reactions without taxonomic and functional annotation from the universal database mentioned above. In particular, we used the FastCoreWeighted implementation from the COBRA Toolbox^38,39^. This algorithm requires the definition of a core, which represents a set of target reactions that must be functional in the final model. We applied the algorithm sequentially for each phenolic compound, defining the core equal to the reactions present in AGREDA^17^ plus the reactions predicted by RetroPath RL^27^.

Finally, we integrated AGREDA^17^ and the reactions FastCoreWeigthed^38^ added to the core at each iteration. Since the algorithm might have added some reactions without any taxonomical information, we removed them and applied fastFVA^32^ to eliminate blocked reactions. Additionally, we applied a single-species analysis, as done in the AGREDA reconstruction^17^, in order to avoid possible dead-end metabolites in the metabolic model of each organism and include transport reactions if we have sufficient evidence for them. Next, we applied fastFVA^32^ to the metabolic model of each organism involved in AGREDA and eliminated blocked reactions. At the end of the entire process, we were able to introduce in the reconstruction 80 out of the 86 phenolic compounds whose degradation was predicted by RetroPath RL^27^. In total, we added 133 metabolites and 313 reactions to AGREDA^17^, obtaining a final network made up of 2735 metabolites and 6257 reactions, which we call AGREDA_1.1.

### Metabolic capabilities of AGREDA in different contexts

For the various analyses conducted in the Results section, in contrast to our previous work^17^, where a mixed-bag network community model was used, we built a compartmentalized network community model with the different versions of AGREDA. In these community models, each species is considered as an independent compartment and the metabolite exchange between different species can be captured. Flux Variability Analysis (FVA) was applied to characterize the metabolic capabilities of the human gut microbiota in different contexts^32^. Particularly, we focus on elucidating different output microbial metabolites derived from the diet.

### In vitro digestion-fermentation of lentils

Lentils were submitted to in vitro digestion^40^ and fermentation^39–42^ resembling the physiological processes along the gastrointestinal tract. Four groups of children (lean, obese, celiac and allergic to cow’s milk) were used as fecal donors to check the effect of different kinds of gut microbiotas.

Regarding in vitro digestion, 5 g of sample were weighed into a screw-cap 50 mL tube. In vitro digestion consisted of three steps: oral, gastric and intestinal. Five milliliters of simulated salivary fluid with 150 U/mL of alpha-amylase were added and mixed into the 50 mL tube carrying the sample and kept at 37 °C for 2 min. Secondly, 10 mL of simulated gastric fluid with 4000 U/mL of gastric pepsin were added to the mix, the pH lowered to 3 and kept at 37 °C for 2 h. Enzyme activity was halted by immersion in ice for 15 min. Tubes were centrifuged, the supernatant (fraction available for absorption at the small intestine) collected and the pellet (fraction not digested that would reach the colon) used for in vitro fermentation. Salt composition of simulated fluids can be found in Supplementary Table 6.

Fecal samples from three donors of each children population (8–10 years old, 95 % percentile and they had not taken antibiotics in the last three months) were used for the in vitro fermentation. Common exclusion criteria were diagnosis of chronic gastrointestinal disorders or any other chronic disease or special diet other than those specific for celiac or allergic children, as well as having taken antibiotics or probiotics three months before the start of the study. Recruitment of the study participants was done via the pediatric unit at the hospital in Athens (Greece). Parents were given an informed consent as well as information and questionnaires for inclusion/exclusion criteria. The study was approved by ethics committee at the University General Hospital in Athens.

Fecal material was pooled by donor group (lean, celiac, allergic and obese children) to account for inter-individual variability. In vitro fermentation was carried out at 37 °C for 20 h, in oscillation. For this purpose, 0.5 g of the pellet obtained after in vitro gastrointestinal digestion were used, as well as 10% of the supernatant. Fermentation medium composed of peptone (14 g/L, cysteine 312 mg/L, hydrogen sulfide 312 mg/L and resazurin 0.1% v/v) was added to the fermentation tube at a volume of 7.5 mL. A fecal inoculum was made from fecal material by mixing it with PBS at a concentration of 33%. Two milliliters of inoculum were added to the fermentation tube. Afterwards, nitrogen was bubbled into the tube until reaching anaerobic conditions (transparent solution as opposed to pink when oxygen is dissolved). After 20 h at 37 °C, microbial activity was halted by immersion in ice for 15 minutes and tubes were centrifuged to collect the supernatant (fraction available for absorption at the large intestine), which was stored at −80 °C until further analysis. Blanks carrying water instead of sample were included in the in vitro digestion as well as in the in vitro fermentation.

### Untargeted metabolomics

Fermented extracts were filtered prior to UPLC injection (2.5 μL). A quality control sample was randomly prepared and injected during analysis. This control was performed to attenuate the resulting analytical variation and to monitor the stability of the system.

MassLynx v4.1. software was used to control the complete system. The system included a time of flight-mass spectrometer detector (SYNAP G2 from Waters Corp., Milford, MA, USA) coupled to LC equipment ACQUITY UPLC M-Class System (Waters Corp., Milford, MA, USA). The UPLC column used was a Poroshell 120, SB-C18 (Agilent Technologies, Palo Alto, CA, USA). The mobile phases used were A acidified water and mobile phase B acetonitrile. A linear gradient was applied maintaining a fixed flow rate of 0.6 mL/min and 25 °C throughout the gradient. Mass spectrometry (MS) analyses were carried out in full-scan mode using an electrospray interface. All MS data were acquired using LockSpray to ensure mass accuracy and reproducibility. The molecular masses of the product ions and precursor ion were accurately determined with leucine encephalin.

Raw data were processed with MassLynx v4.1 software (Waters, USA) according to the “find-by-formula” algorithm. To achieve a higher confidence in metabolite identification, the spectral isotope pattern was used together with accurate mass information. The data were analyzed based on their coefficient of variation with the quality-control sample. Phenol-Explorer 3.6 and Human Metabolome Database were used as references for compound identification. The identification was carried out as established by the COSMOS Metabolomics Standards Initiative (http://cosmos-fp7.eu/msi). Finally, potential metabolites that exceeded the mass accuracy detection threshold, showed significantly different trends from the control (fecal fermentation without lentils) and had plausible peak characteristics in the chromatogram were considered as possible fermentation markers for the different conditions.

## Supplementary information

Supplementary Material

Supplementary Tables

Supplementary Data 1

Supplementary Data 2

## Data Availability

The 16 S rRNA sequencing data were obtained from https://www.ebi.ac.uk/ena/browser/home under accession code PRJEB40603, being summarized in Supplementary Table 4. The metabolomics data are provided in Supplementary Table 5. The rest of the data employed in this study can be obtained from the following databases: (i) Metabolic models: AGORA (https://www.vmh.life/), The Model SEED (https://modelseed.org/), AGREDA (https://github.com/tblasco/AGREDA); (ii) Metabolites and Chemical rules: PubChem (https://pubchem.ncbi.nlm.nih.gov/), Human Metabolome Database (https://hmdb.ca/), RetroRules (https://retrorules.org/), i-Diet (http://www.i-diet.es/), Phenol-Explorer (http://phenol-explorer.eu/), MolDB (https://moldb.wishartlab.com/). The source data underlying Figs. 2a-c, 3a and c, and 4 are provided as a Source Data file.

## Code availability

The source code to generate AGREDA_1.1 can be found in https://github.com/francesco-balzerani/AGREDA_1.1.
