# Supplementary material for: Prediction of degradation pathways of phenolic compounds in the human gut microbiota through enzyme promiscuity methods
Source: NPJ Syst Biol Appl. 2022 Jul 12;8:24. doi: 10.1038/s41540-022-00234-9 (PMC9279433; doi:10.1038/s41540-022-00234-9)
Supplement: Supplementary file 1 — Supplementary Material [file 41540_2022_234_MOESM1_ESM.pdf]

Supplementary Information: Prediction of degradation pathways of phenolic compounds in the human gut microbiota through enzyme promiscuity methods

Francesco Balzerani[1], Daniel Hinojosa-Nogueira[4,a], Xabier Cendoya[1,a], Telmo Blasco[1], Sergio Pérez-Burillo[4], Iñigo Apaolaza[1,2,3], M. Pilar Francino[5,6], José Ángel Rufián-Henares [4,7,*], and Francisco J. Planes[1,2,3*]

[1]University of Navarra, Tecnun School of Engineering, Manuel de Lardizábal 13, 20018 San Sebastián, Spain.

[2]University of Navarra, Biomedical Engineering Center, Campus Universitario 31009 Pamplona, Navarra, Spain.

[3]University of Navarra, Instituto de Ciencia de los Datos e Inteligencia Artificial (DATAI), Campus Universitario, 31080, Pamplona, Spain

[4]Departamento de Nutrición y Bromatología, Instituto de Nutrición y Tecnología de los Alimentos, Centro de Investigación Biomédica, Universidad de Granada, Granada, Spain.

[5]Area de Genómica y Salud, Fundación para el Fomento de la Investigación Sanitaria y Biomédica de la Comunitat Valenciana-Salud Pública, Valencia, Spain.

[6]CIBER en Epidemiología y Salud Pública, Madrid, Spain

[7]Instituto de Investigación Biosanitaria ibs.GRANADA, Universidad de Granada, Granada, Spain.

[a]*Equal contribution*

[*]*Corresponding authors: fplanes@tecnun.es; jarufian@ugr.es*

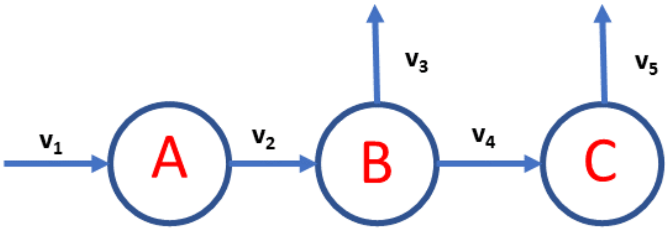

Supplementary Figure 1: Example metabolic model that explicates the underlying under-determination of constraints-based models. This example metabolic network is made of: 3 metabolites (A, B, C) and 5 reactions whose fluxes are defined as v1, v2, v3, v4 and v5. Assuming that the input flux of A is non-zero (v1>0), the reaction v2 (>0) leads to the production of B. At this point, B may be excreted through the exchange reaction v3 (>0) or transformed in C via the reaction v4 (>0). In the same way, once C is produced it can be excreted through the exchange reaction v5 (>0). In our computational approach, we calculate the maximum exchange flux for each output metabolite, and, as a result, we predict both B and C may be potentially produced. However, it may happen that just one of them is produced. A real-life scenario in which this can happen is when an intermediate metabolite (B in Supplementary figure 2) is quickly transformed into another one (C in this case) and, therefore, it is not present in the sample, even though it was actually produced because is needed to obtain C. Without additional -omics data, we cannot distinguish and correctly characterize these cases. This underdetermination can be observed in the high number of false positives for few predicted output metabolites in the lentil fermentation study, *e.g.* protocatechualdehyde (see Figure 4 in the main text).

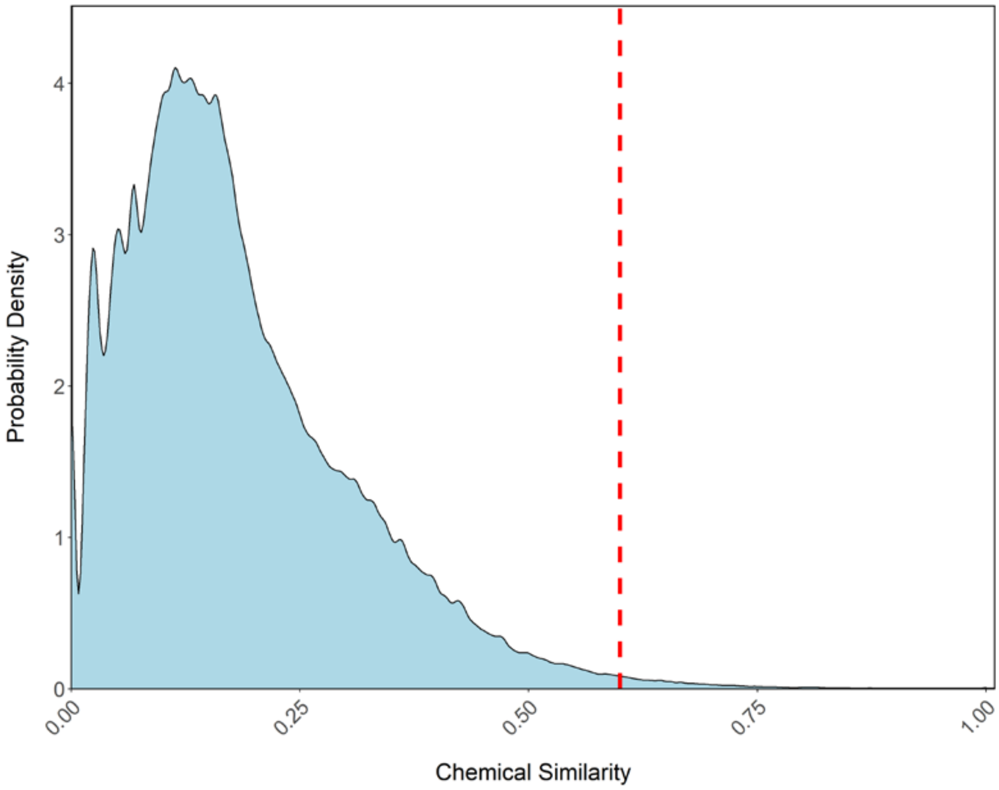

Supplementary Figure 2: Distribution of chemical similarity between source compounds and reaction rule's substrates. RetroPath RL[1] provides a parameter defining a chemical cut-off value related to the similarity between the molecule of interest and the substrate of the template to limit the application of the algorithm. In order to define that value, we computed the similarity between source compounds and reaction rule's substrate, using the rdkit package[2] and the Morgan fingerprint[3] with a radius equal to 2. In the representation of the similarity values distribution is marked in red the selected threshold at 0.6. Since more than 97% is below this cut-off value, proper analysis of the metabolic space is permitted not introducing excessive promiscuity. Source Data is provided as a Source Data file.

Supplementary Note 1: Manual curation of predicted reactions by RetroPath RL

Change of the stoichiometry of annotated reactions. In the process of balancing the predictions by RetroPath RL[1], we found out that the predictions related to the reaction below (obtained from literature and included in AGREDA_1.0[4]) were inconsistent at the stoichiometric level. Therefore, we revised this reaction and its corresponding predictions.

Mirtillin -> D-glucose + Gallate + Phloroglucinol

The reaction under analysis is a multistep reaction, which can be divided into 3 parts: deglycosylation, C-ring fission and dehydroxylation[5], and decarboxylation and methyl transference, namely:

1. Deglycosylation:

   Mirtillin + Water -> Delphinidin + D-glucose

2. C-ring fission and dihydroxylation:

   Delphinidin + Water -> 3,5-Dihydroxyphenylacetic acid + Gallate + H

3. Decarboxylation and methyl transference:

   3,5-Dihydroxyphenylacetic acid + H2O -> Phloroglucinol + CO2 + Methane

In summary, the complete reaction should read:

Mirtillin + Water -> D-glucose + Gallate + Phloroglucinol + H + CO2 + Methane

We observed that, with the first step of this reaction, source compounds could directly reach several sink compounds. Therefore, when at the first step of the pathway it was possible to create metabolites already present in the reconstruction, we changed the prediction with that transformation, avoiding the complexity of the multistep pathway. Instead, in case it was not possible, the whole balanced pathway was introduced replacing the original prediction. Following the correct balance of the multistep reaction, the template was also changed in both AGREDA_1.0[4] and AGREDA_1.1.

Revising output metabolites in the predicted reactions. After the balancing process, we carried out a manual revision of the results of RetroPath RL[1]. In some of the predicted reactions, we noticed that the product metabolites were incorrectly substituted with other molecules of high similarity. One example involved a glucoside group where the *D-Glucose* is broken out, but the products included *D-Galactose* instead.

As a verification step, we calculated the pairwise similarity between the predicted metabolites and the ones that, according to our curation, would make more sense in the given reaction. In order to do so, we used the circular fingerprint[3] of the rdkit[2] package in python. The value of the similarity was equal to 1.0 in all cases, meaning that the algorithm was unable to make a difference between them. Given this limitation of RetroPath RL[1], we manually curated the equations involving sugars and replaced the predicted outputs with the appropriate ones.

Resolving predicted metabolites. In several cases the predicted reactions had compounds marked as present in the sink, but there was a lack of information, being written just with their InChI Key ID. For each of them, a similarity analysis with the metabolite in the sink was carried out. For some compounds the similarity returned more than one positive result. In those cases, we selected the most coherent compound with the predicted transformation.

In other situations, the predicted reactions had intermediate compounds not present in the sink, being only provided with their chemical structure. A manual curation in several databases was carried out. We updated the information of the compounds present in databases and removed those molecules with no information and their associated reactions.

New input exchange fluxes. We introduced an input exchange reaction for the metabolite *1-Feruloyl-D-glucose*. This compound is a key component in some reactions that RetroPath RL[1] predicted, but its production was possible only using reactions without any associated taxonomy. In order to avoid this type of reactions, we added the input exchange reaction for

such metabolite. It was possible to introduce this input because the metabolite under analysis can be assimilated through the diet, since it is found in different fruits of vegetables[6-7].

Reaction directionality definition. Inconsistent reaction directionality was found during the analysis of the results. Some of the newly defined metabolic reactions that were found for phenolic compounds by RetroPath RL[1] happened to be the same as irreversible reactions already contained in the universal database generated with our previous methods[4], but using the opposite direction of the versions contained in that database. This issue stemmed from: a) the differential definitions of reaction directionality contained in the RetroRules[8] with respect to the universal database that we created, and b) RetroRules contains reversed rules to apply on retrosynthesis applications[8]. While this second reason is part of how the application works, we studied reaction directionality consistency in order to ensure that the initial databases that were used to create the rules used by RetroPath RL[1] made sense.

We compared the data of origin that each of these two databases used to define the reactions. On the one hand, RetroRules[8] extracted the metabolic reactions from MNXref 3.0[9] to generate reaction rules in the SMARTS format. The data contained in MNXref[9] itself comes from a collection of Genome-Scale Metabolic Networks that have been reconciled for consistent metabolite, reaction and protein information by comparing them with information from various sources[10–19]. These sources include The Model SEED[13] and Recon3D[20]. The Model SEED[13] was one of the sources that was used to create our universal database, which ensured consistency with our rules. On the other hand, our other large source of metabolic information, AGORA[21], defined reaction directionality using the same Gibbs Free energy estimation carried out for Recon3D[22]. While these estimations have greatly reduced errors[22], their calculations are dependent on environmental conditions, which can vary across reconstructions. MNXref[9], which acts as the base for RetroRules[8], provides reactions that have been reconciled[9] across databases, and thus will not always provide reaction directions that are consistent with the original metabolic networks. Given this, we accept the new reactions

even though their directionality might not match with the universal database that we started with.

References

1. Koch, M., Duigou, T. & Faulon, J. L. Reinforcement learning for bioretrosynthesis. *ACS Synth. Biol.* **9**, 157–168 (2020).

2. Landrum, G. RDKit : A software suite for cheminformatics , computational chemistry , and predictive modeling. *Components* (2011).

3. Rogers, D. & Hahn, M. Extended-connectivity fingerprints. *J. Chem. Inf. Model.* **50**, 742–754 (2010).

4. Blasco, T. *et al.* An extended reconstruction of human gut microbiota metabolism of dietary compounds. *Nat. Commun.* **12**, 1–12 (2021).

5. Marín, L., Miguélez, E. M., Villar, C. J. & Lombó, F. Bioavailability of dietary polyphenols and gut microbiota metabolism: Antimicrobial properties. *Biomed Res. Int.* **2015**, (2015).

6. Du, Q. *et al.* Antioxidant constituents in the fruits of Luffa cylindrica (L.) Roem. *J. Agric. Food Chem.* **54**, 4186–4190 (2006).

7. Arnaldos, T. L., Muñoz, R., Ferrer, M. A. & Calderón, A. A. Changes in phenol content during strawberry (Fragaria x ananassa, cv. Chandler) callus culture. *Physiol. Plant.* **113**, 315–322 (2001).

8. Duigou, T., Du Lac, M., Carbonell, P. & Faulon, J. L. Retrorules: A database of reaction rules for engineering biology. *Nucleic Acids Res.* **47**, D1229–D1235 (2019).

9. Moretti, S. *et al.* MetaNetX/MNXref - Reconciliation of metabolites and biochemical reactions to bring together genome-scale metabolic networks. *Nucleic Acids Res.* **44**, D523–D526 (2016).

10. Schellenberger, J., Park, J. O., Conrad, T. M. & Palsson, B. Ø. BiGG: a Biochemical Genetic and Genomic knowledgebase of large scale metabolic reconstructions. *BMC Bioinformatics* **11**, (2010).

11. Caspi, R. *et al.* The MetaCyc database of metabolic pathways and enzymes. *Nucleic Acids Res.* **46**, D633–D639 (2018).

12. Büchel, F. *et al.* Path2Models: Large-scale generation of computational models from biochemical pathway maps. *BMC Syst. Biol.* **7**, (2013).

13. Overbeek, R. *et al.* The SEED and the Rapid Annotation of microbial genomes using Subsystems Technology (RAST). *Nucleic Acids Res.* **42**, 206–214 (2014).

14. Kim, H. *et al.* YeastNet v3: A public database of data-specific and integrated functional gene networks for Saccharomyces cerevisiae. *Nucleic Acids Res.* **42**, 731–736 (2014).

15. Kanehisa, M. & Goto, S. KEGG: Kyoto Encyclopedia of Genes and Genomes. *Nucleic Acids Res.* **28**, 27–30 (2000).

16. Forster, M., Pick, A., Raitner, M., Schreiber, F. & Brandenburg, F. J. The System

Architecture of the BioPath System. *In Silico Biol.* **2**, 415–426 (2002).

17. Croft, D. *et al.* The Reactome pathway knowledgebase. *Nucleic Acids Res.* **42**, 472–477 (2014).

18. Morgat, A. *et al.* Updates in Rhea-a manually curated resource of biochemical reactions. *Nucleic Acids Res.* **43**, D459–D464 (2015).

19. Morgat, A. *et al.* UniPathway: A resource for the exploration and annotation of metabolic pathways. *Nucleic Acids Res.* **40**, 761–769 (2012).

20. Brunk, E. *et al.* Recon3D enables a three-dimensional view of gene variation in human metabolism. *Nat. Biotechnol.* **36**, 272–281 (2018).

21. Magnúsdóttir, S. *et al.* Generation of genome-scale metabolic reconstructions for 773 members of the human gut microbiota. *Nat. Biotechnol.* **35**, 81–89 (2017).

22. Noor, E., Haraldsdóttir, H. S., Milo, R. & Fleming, R. M. T. Consistent Estimation of Gibbs Energy Using Component Contributions. *PLoS Comput. Biol.* **9**, (2013).
